# A network approach to decentralized coordination of energy production-consumption grids

**DOI:** 10.1371/journal.pone.0191495

**Published:** 2018-01-24

**Authors:** Elisa Omodei, Alex Arenas

**Affiliations:** Departament d’Enginyeria Informàtica i Matemàtiques, Universitat Rovira i Virgili, 43007 Tarragona, Spain; Universita degli Studi della Tuscia, ITALY

## Abstract

Energy grids are facing a relatively new paradigm consisting in the formation of local distributed energy sources and loads that can operate in parallel independently from the main power grid (usually called microgrids). One of the main challenges in microgrid-like networks management is that of self-adapting to the production and demands in a decentralized coordinated way. Here, we propose a stylized model that allows to analytically predict the coordination of the elements in the network, depending on the network topology. Surprisingly, almost global coordination is attained when users interact locally, with a small neighborhood, instead of the obvious but more costly all-to-all coordination. We compute analytically the optimal value of coordinated users in random homogeneous networks. The methodology proposed opens a new way of confronting the analysis of energy demand-side management in networked systems.

## Introduction

Local distributed energy production-consumption nodes (microgrids) are becoming an important complement, and even alternative, to centralized energy generation facilities. Energy transmission grids have been largely analyzed in the complex networks literature to assess performance, efficiency and robustness [1–13].

However, the analysis of microgrids present new challenges rooted in its own idiosyncrasy of functioning, in particular the problem of distributed control [14–18]. Users in a microgrid can be understood as nodes of a production-consumption network operating, ideally, disconnected from the main electric grid. A microgrid can be powered by distributed generators, batteries, and/or renewable resources like solar panels. Depending on how it is charged and how its requirements are managed, a microgrid might run alone indefinitely. The main problem in this set up is to achieve a good balance between production and consumption in such a way that users are always coordinated in the use of the available energy. The scenario depicted can be abstracted to the mathematical problem of assessing local coordination of networked agents having access to the information of the total energy production in time. Note that total production can be simply broadcasted to all individuals, however global coordination is a costly and difficult interplay.

Here we take advantage of the theory of complex networks to propose a stylized model for coordinating the different users’ consumption demands according to the energy produced within the microgrid. Our results show that local coordination can effectively provide a way to satisfy users’ demands restricted to the available energy production. The model, for random homogeneous networks, raises interesting results, attaining very large coordination levels (more than 95% of users) for relatively low number of coordinating neighbors (usually 10^−3^N, being N the number of users).

## Results

### A coordination model for decentralized production-consumption control

**Fig 1:**
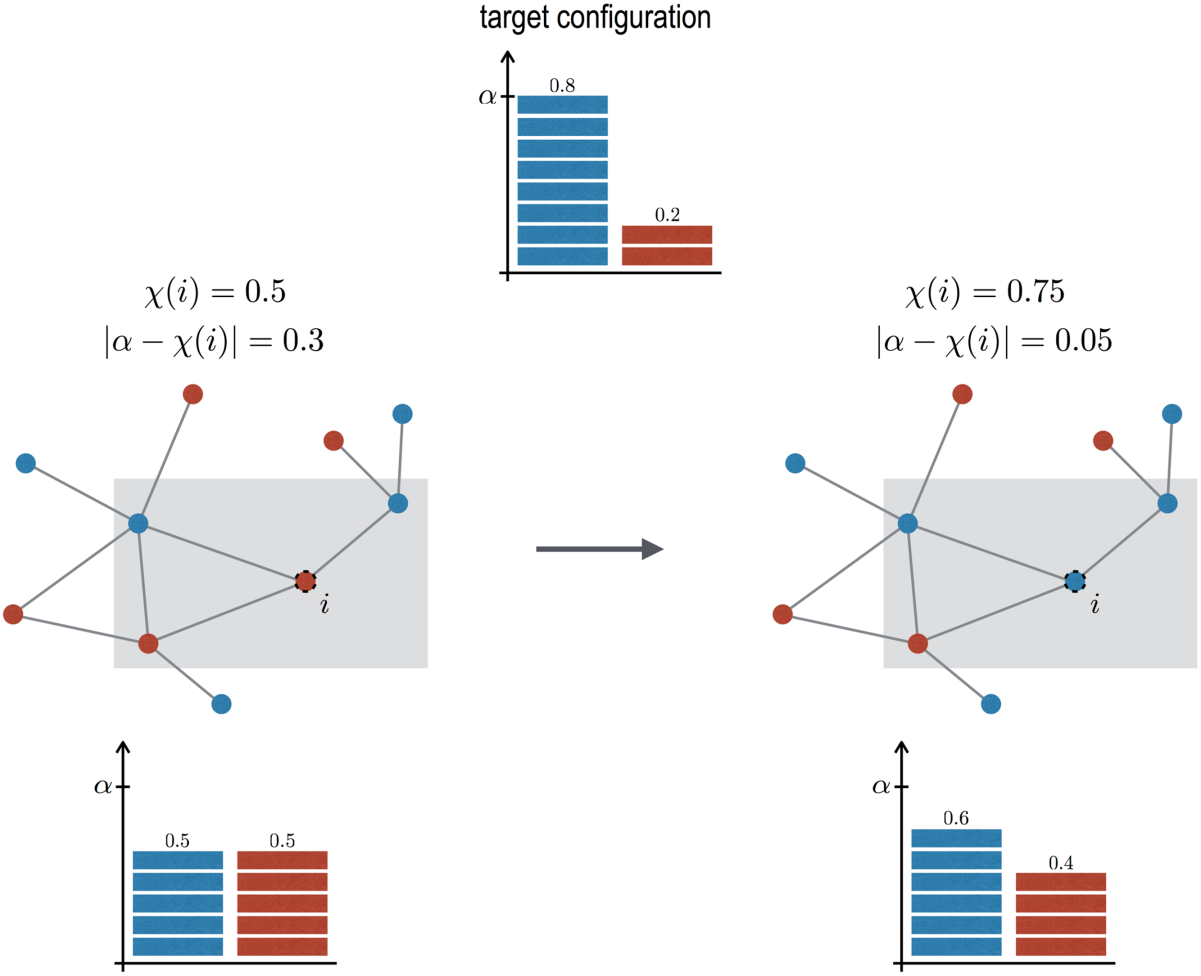
Model illustration. We consider a simple network of *N* = 10 users, where nodes colored in blue represent users in the *on* state, whereas red represents the *off* state. The top histogram shows the target configuration, in which *αN* users are in the *on* state. In the left network, half users are *on*, as represented in the corresponding histogram. user *i* is randomly selected. The local configuration centered on it is also composed of half *on* users (*χ*(*i*) = 0.5), and therefore |*α* − *χ*(*i*)| = 0.3. If user *i* switches *on*, then the local configuration will consist of 3 *on* users out of 4 (*χ*(*i*) = 0.75), and the local difference with the target configuration *α* decreases to 0.05. Therefore the user switches *on*, improving also the global configuration, that becomes closer to the target, as shown in the bottom right histogram.

We consider a population of *N* networked users representing the users of the microgrid. To simplify the users’ consumption activity, each user *i* is characterized by a binary variable λ_*i*_ that denotes its state, which can be either *on* (1) or *off* (0), for *i* = 1…*N*. The control goal is for the system to reach a “target” configuration, characterized by the parameter *α* (0 ≤ *α* ≤ 1), which represents the fraction of users that should be *on*, that is the consumption (load) that can be attained given the energy production. This is not the physical synchronization of the nodes of the underlying electrical grid, but a measure of how many users should concurrently consume electricity in order to match the available energy. To reach this target configuration we propose the dynamical model illustrated in Fig 1. At each time step, an user *i* is randomly selected among the population. The user only has partial information on the state of the system, that is it only sees its own state and the state of its neighbors. This information provides the local configuration of the system, centered on user *i*,

$$\chi \left(i\right)=\frac{\lambda_{i}+\sum_{n\in N(i)}\lambda_{n}}{1+k_{i}}$$ (1)

where *k*_*i*_ is the user’s degree (i.e. the number of neighbours), and N(i) its neighbors set. The user compares the local configuration *χ*(*i*) with the target configuration *α*. If they differ, then it evaluates if changing its own state would reduce the difference between *α* and the new local configuration, that is given by $\hat{\chi }\left(i\right)=\frac{\left(1-\lambda_{i}\right)+\sum_{n\in N(i)}\lambda_{n}}{1+k_{i}}$. If indeed $|\alpha -\hat{\chi }\left(i\right)|<|\alpha -\chi \left(i\right)|$ then the user changes its state λ_*i*_ to 1 − λ_*i*_, otherwise it keeps its state as it is. In the physics literature, the proposed model corresponds to an Ising model in an uniform external field and temperature equal to zero.

### Optimal coordination in random homogeneous networks

We test the previous model on Erdős-Rényi (ER) networks, of size N, with different edge probabilities *p*, that determine the network average degree 〈*k*〉 = *p*(*N* − 1). The network topology defines the “social” neighbors each user coordinates with, and does not need to correspond with the underlying distribution network users’ households are connected to. Even though ER networks do not reproduce the main characteristics of empirical social networks, the interest here is to investigate the minimum number of neighbors users need to coordinate with in order for the target configuration to be achieved. Coordination could then take place online, via a dedicated app, therefore the proposed social structure does not need to correspond to observed online and offline social networks.

We start from an initial configuration in which states are randomly assigned so that approximately half of the users are *on*, and half are *off*, and we run the dynamics described in the previous section until the system reaches an stationary state. To represent the coordination level *C* of users with the energy production, we define

$$C=1-|\alpha -\frac{1}{N}\sum_{i=1}^{N}\lambda_{i}|\;\text{.}$$ (2)

We also define a second measure, that we will call alteration level *A*, indicating the fraction of users that have changed their state according to the model

$$A=\frac{1}{N}\sum_{i=1}^{N}\left|\lambda_{i}\left(t_{\infty }\right)-\lambda_{i}\left(t_{0}\right)\right|.$$ (3)

The lower the alteration the better in terms of users’ initial requirements.

**Fig 2:**
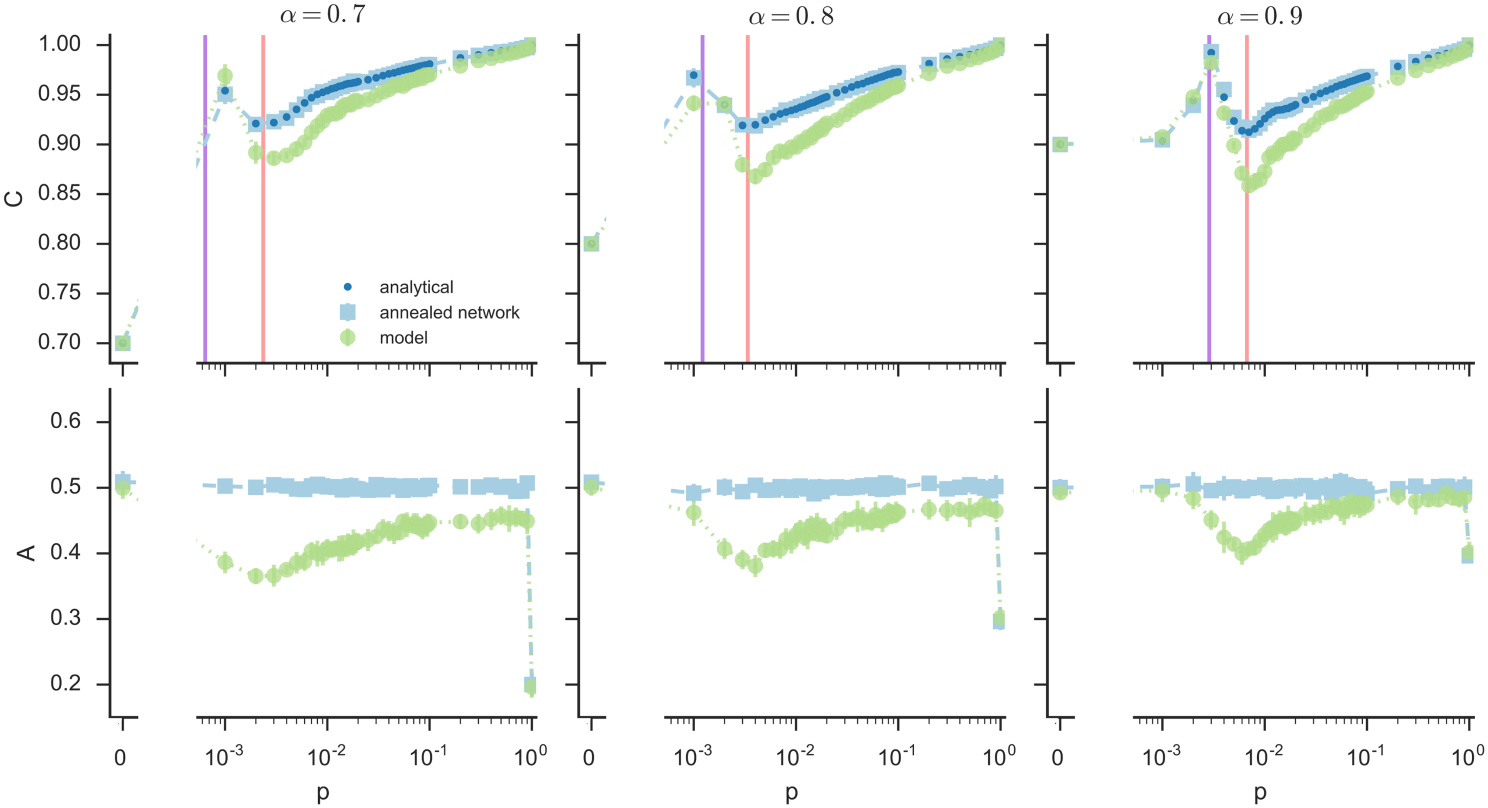
Final coordination and alteration levels in function of the network edge probability *p*, for different values of the target configuration *α*. The three top panels show, for three different values of *α*, the coordination level at the stationary state in function of the graph edge probability *p*. The corresponding bottom panels show the alteration level. Green circles represent the model results, blue points represent the mean-field analytical values, and light blue squares represent the results of the randomized neighbors model, in which at each time step a randomly selected user *i* interacts with a different set of *k*_*i*_ random users in the populations. The purple line represents the point in which the coordination level takes its maximum in the 0 < *p* < 1 region according to the analytical calculations, and the red line represents the point in which it takes its minumum. For each *p*, we run the models on 10 different Erdős-Rényi graphs *G*(*N*, *p*), with *N* = 1000, initially assigning to each user the *on* or *off* state with equal probability 0.5. The points shown represent the averaged results, and error bars represent the standard deviation from the mean.

The results of the simulations are reported in Fig 2, green circles. The three top panels show, for different values of *α*, the system coordination level at the stationary state. The three bottom panels show the system alteration level, that is the fraction of users that at the stationary state have a different state with respect to the initial configuration. We show results only for *α* > 0.5 because, thanks to symmetry, for each value of *α* < 0.5 the results are the same as for 1 − *α*.

The results reveal that it exists a value of the connectivity, represented by the probability *p* of connecting two nodes, for which the coordination is almost complete and the alteration is minimal (for *p* < 1). For this value, the target configuration is reached with the least effort (i.e. the least change in the users’ states) and less communication. The result is only improved at a larger cost when *p* = 1, that is when each user is connected to every other user in the network, because in this topology users have complete information on the state of the system.

This surprising result can be explained by noticing that when the local configuration *χ*(*i*) only includes a few users, which is what happens when *p* approaches 0, then *on* states are favored. This is true when *α* > 0.5. If *α* < 0.5 the following reasoning can be reversed symmetrically for *off* states. Consider for example an isolated user (*k*_*i*_ = 0). For any *α* > 0.5, *χ*(*i*) is either 1 or 0 (if the user is *on* or *off*, respectively), therefore the user always prefers the *on* state, because |*α* − 1| < |*α* − 0|. Moreover, for an user with only one neighbor (*k*_*i*_ = 1), *χ*(*i*) can be equal to 1 (if both users are *on*), 0.5 (if one is *on* and the other is *off*), or 0 (if both are *off*). Therefore if *α* > 0.75, the user turns *on* (or remains *on* if it already was) for any value of its neighbor’s state, because this is what minimizes |*α* − *χ*(*i*)| in all cases. This reasoning can be extended for higher values of *k*, up to a point in which *k* is large enough so that *χ*(*i*) can take enough different values for the discretization not to bias the choice of the state. Therefore, below a critical *p* (whose value depends on *α*), the fraction of users that at the stationary state are *on* starts increasing while *p* decreases, reaching 1 when *p* = 0. Correspondingly, the coordination level defined by Eq 2 equals *α* when *p* = 0 and the fraction of *on* users is 1. It then increases with *p* while the fraction of *on* users decreases approaching the target state *α*, until a point in which this fraction becomes smaller than *α*, after which the coordination level starts decreasing as the configuration starts distancing the target state on the opposite direction (that is, before the maximum there were too many *on* users, after it there are too few). After reaching a minimum at the critical value of *p* for which discretization does not favor *on* states anymore, the coordination level starts increasing again while *p* increases (since more and more information available to the users), reaching 1 when *p* = 1.

For every *α* considered, we can identify some small value of *p* for which we have both high coordination and low alteration, showing that, reducing the number of users that have to coordinate can actually improve the whole system coordination in random homogeneous networks.

### Mean-field approach

In this section we derive the mean-field analytical calculation of the coordination level for a given *p*, and then find the critical values of *p* for which coordination takes its minimum and maximum (in the 0 < *p* < 1 region). Let us denote with *U*(*t*) the number of users that at time *t* are *on*. This quantity evolves over time according to the following equation:

$$U\left(t+1\right)=U\left(t\right)+p_{01}\left(p,U\left(t\right)\right)\frac{N-U(t)}{N}-p_{10}\left(p,U\left(t\right)\right)\frac{U(t)}{N}$$ (4)

where *p*_01_(*p*, *U*(*t*)) is the probability of an user to switch its status from 0 to 1 and *p*_10_(*p*, *U*(*t*)) the probability of switching from 1 to 0. The former probability is multiplied by the probability of finding an *off* user in the population, that is $\frac{N-U(t)}{N}$, and the latter by the probability of finding an *on* user, that is $\frac{U(t)}{N}$. According to the model presented, the two switching probabilities follow the binomial distribution:

$$p_{01}\left(p,U\left(t\right)\right)=\sum_{k=0}^{N-1}\left(\begin{array}{c} N-1\\ k \end{array}\right)p^{k}{\left(1-p\right)}^{N-1-k}f\left(U\left(t\right),k\right)$$ (5)

$$p_{10}\left(p,U\left(t\right)\right)=\sum_{k=0}^{N-1}\left(\begin{array}{c} N-1\\ k \end{array}\right)p^{k}{\left(1-p\right)}^{N-1-k\;}\bar{f}\left(U\left(t\right),k\right)$$ (6)

where the distribution of users in the *on state*, *f*(*U*(*t*), *k*), *and in the* off state, $\bar{f}\left(U\left(t\right),k\right)$, follow the hypergeometric distribution:

$$\begin{array}{c} f\left(U\left(t\right)\right)=\sum_{\nu =\text{max}\left(0,k+U\left(t\right)-N\right)}^{\text{min}\left(k,U\left(t\right)\right)}\frac{\left(\begin{array}{c} U\left(t\right)\\ \nu \end{array}\right)\left(\begin{array}{c} N-1-U\left(t\right)\\ k-\nu \end{array}\right)}{\left(\begin{array}{c} N-1\\ k \end{array}\right)}[1-\theta (\left|\alpha -\frac{1+\nu }{1+k}\right|-\left|\alpha -\frac{\nu }{1+k}\right|) \end{array}$$ (7)

$$\begin{array}{c} \bar{f}\left(U\left(t\right)\right)=\sum_{\nu =\text{max}\left(0,k+\left(U\left(t\right)-1\right)-\left(N-1\right)\right)}^{\text{min}\left(k,U\left(t\right)-1\right)}\frac{\left(\begin{array}{c} U\left(t\right)-1\\ \nu \end{array}\right)\left(\begin{array}{c} N-1-\left(U\left(t\right)-1\right)\\ k-\nu \end{array}\right)}{\left(\begin{array}{c} N-1\\ k \end{array}\right)}[1-\theta (\left|\alpha -\frac{\nu }{1+k}\right|-\left|\alpha -\frac{1+\nu }{1+k}\right|)] \end{array}$$ (8)

and *θ*(*x*) is the Heavside step function, which is 0 if *x* < 0, and 1 otherwise.

At the stationary state, Eq 4 becomes

$$\begin{array}{c} p_{01}\left(p,U^{\infty }\right)\left(N-U^{\infty }\right)-p_{10}\left(p,U^{\infty }\right)U^{\infty }=0\;\text{.} \end{array}$$ (9)

Solving this equation numerically as a fixed point equation, we obtain *U*^∞^, that is the number of *on* users at the stationary state, and then we use it to compute the coordination level, that is given by $1-|\alpha -\frac{U^{\infty }}{N}|$. The results are shown in Fig 2 as blue dots. Note that the results of the mean-field approach present the exact qualitative behavior of the numerical simulations, with a shift that comes from the neglected correlations, having the same optimal values of *p*^†^ and p* for which minimum and maximum coordination is attained, respectively. The value of *p*^†^ corresponding to the minimal coordination (red line) can be computed by solving the following system of equations:

$$\{\begin{array}{c} U^{\infty }=g\left(p,U^{\infty }\right)\\ \frac{\partial g\left(p,U^{\infty }\right)}{\partial p}=0 \end{array}$$ (10)

where

$$\begin{array}{c} g\left(p,U^{\infty }\right)\equiv N\frac{p_{01}\left(p,U^{\infty }\right)}{p_{01}\left(p,U^{\infty }\right)+p_{10}\left(p,U^{\infty }\right)}. \end{array}$$ (11)

The values of p* corresponding to maximum coordination (purple line) can be computed by solving Eq 9 with respect to *p*, having fixed *U*^∞^ = *αN*.

To check the accuracy of the analytical calculations, we run simulations on an annealed random network, which must be coherent with the mean-field approach. Simulations on the anneal network are conducted as follows. Having assigned a degree *k*_*i*_ to each user *i* according to a degree distribution generated by an ER graph *G*(*N*, *p*), we then let the user see a different random subset *k*_*i*_ of the population each time it is selected, instead of the same fixed set of neighbors. The results of this model are shown as light blue squares in Fig 2, and perfectly match the analytical dots, as expected.

For most values of *p*, the coordination level is higher for the annealed version of the model than for the original network. However, not fixing the network structure also implies a higher number of switches during the dynamics, since the local configuration of a node constantly changes. This is reflected in the alteration level, which is always higher for the annealed network, as shown in the bottom panels of Fig 2.

**Fig 3:**
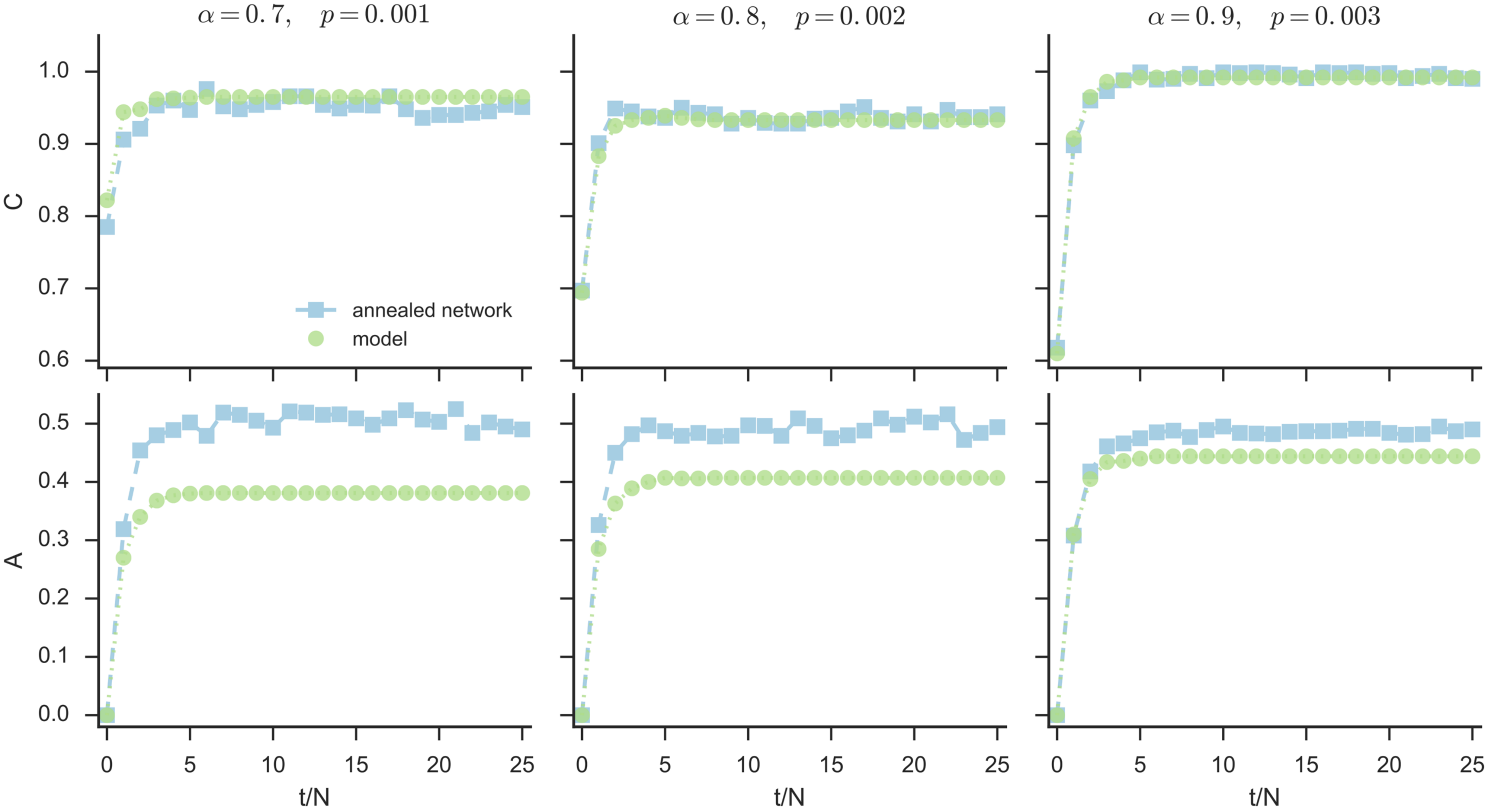
Evolution of coordination and alteration level. The three top panels show, each for a fixed target *α* and graph edge probability *p*, the evolution, every *N* time steps, of the coordination level during the course of one realization of the model dynamics. The three bottom panels show the corresponding evolution of the alteration level. The initial configuration is the one described in Fig 2, and again green circles represent the model results, and light blue squares represent the results of the randomized neighbors model. For each *α*, we plot the results for the dynamics on the ER network with the smallest *p* that maximizes coordination and minimizes alteration (see the corresponding points in Fig 2).

Fig 3 shows the evolution of the coordination and alteration levels over time for both models, for a specific *p*, which is chosen as a function of *α* as the smallest *p* that maximizes coordination and minimizes alteration (see the corresponding points in Fig 2). We can observe that, for these values of *p*, the coordination level is approximatively the same in the two models, whereas alteration is systematically lower in the original model. This indicates that fixing the network topology can lead to the same amount of coordination than in a mean-field situation, but with less effort (i.e. fewer changes in the users’ states).

## Discussion

Let us now discuss how the proposed model can be applied to energy demand side management. Suppose a microgrid which produces a daily amount of energy *E*, that has to be shared among its *N* households. Let us assume that over the whole day the microgrid produces enough energy to sustain the users daily consumption, i.e. the total demand, also known as load *L*, is equal to *E*. Even under this assumption, what it is crucial for the microgrid to work in isolated mode is that production and consumption match *at each time*.

Let us split the day in two parts, that we will simply call, with an abuse of lexicon, morning and afternoon. Imagine that a fraction *α* of *E* is produced in the morning, and the remaining 1 − *α* fraction in the afternoon. Each household in the microgrid is equipped with its own home appliances, that users turn on at different times of the day. Let us focus, as an example, on the washing machine. We suppose that each household wants to run the washing machine once per day, either in the morning or in the afternoon. To reach a matching of energy production and consumption, the system would need *α* per cent of the households to run, for example, the washing machine in the morning, and the other 1 − *α* per cent in the afternoon. This is the same idea at the basis of the proposed model, in which the target configuration is to have *αN* users in the *on* state, and (1 − *α*)*N* *off*. In the microgrid scenario, the target configuration of the model represents the fraction of energy available in the morning, and therefore the expected number of households that should run their washing machines in the morning. The results presented in the previous sections suggest that, in a situation in which no global information is available, a good matching between energy production and consumption can be obtained with very little information, having households coordinating only locally with their closest neighbors.

**Fig 4:**
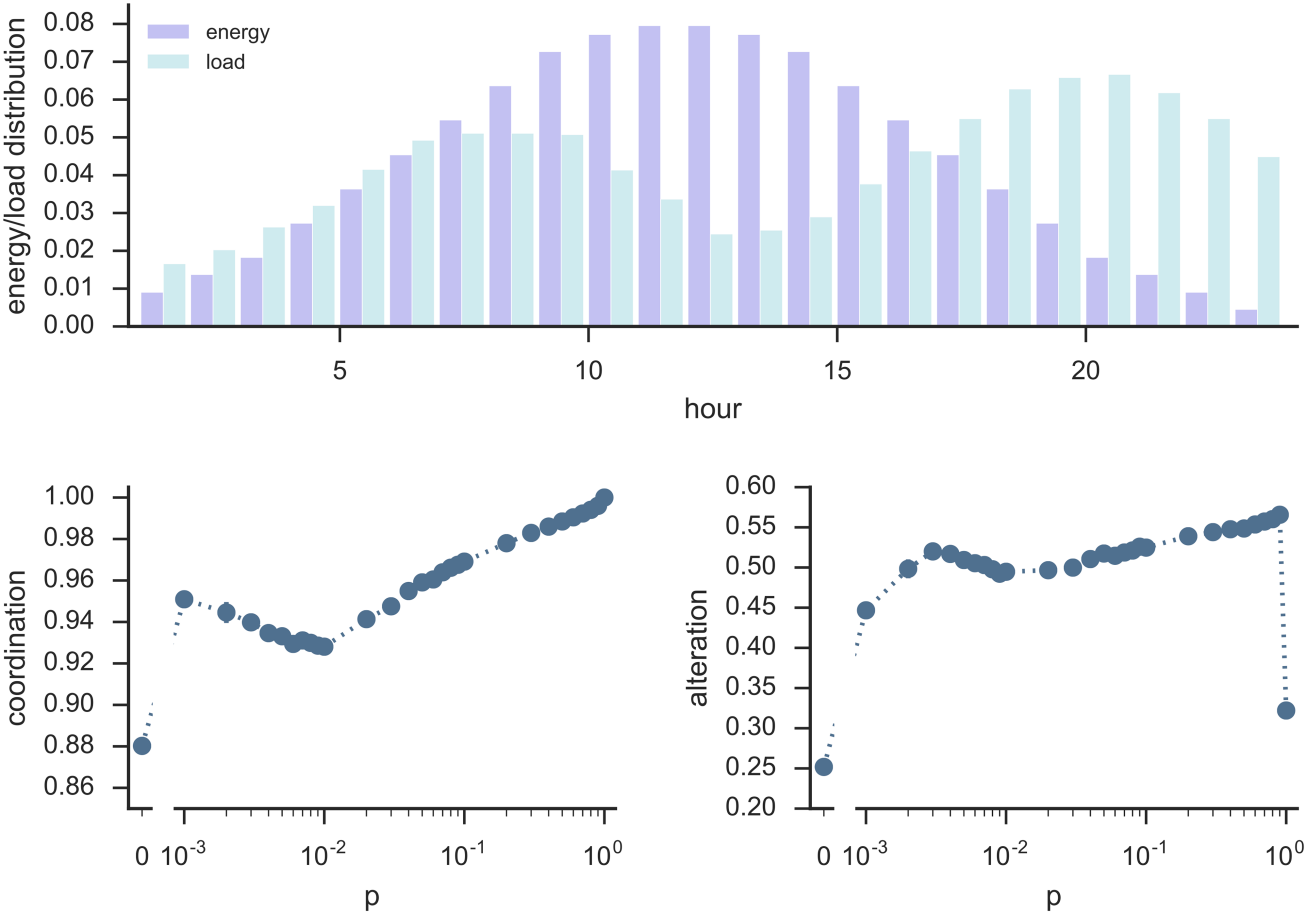
Final coordination and alteration levels in function of the network edge probability *p*, for the extended model simulating demand side management in microgrids. The left bottom panel shows the coordination level at the stationary state in function of the network edge probability *p*, as defined in Eq 14. The right bottom panel shows the corresponding alteration level (Eq 15). For each *p*, we run the models on 10 different Erdős-Rényi graphs *G*(*N*, *p*), with *N* = 1000, initially assigning to each user *i* a state vector ***θ***_*i*_ with 0 and 1 values distributed over the *H* = 24 entries with probabilities drawn from the distribution shown in the top panel (light blue bars). The top panel also shows the target configuration (lilac bars). The points shown in the bottom panels represent the averaged results, and error bars represent the standard deviation from the mean.

The model can be extended to take into account a more realistic scenario in which the day is divided in *H* = 24 parts, that is the 24 hours, and each household has several appliances that might be used during the day. Each user *i* is now characterized, instead that by a scalar variable λ_*i*_, by a vector ***θ***_*i*_ composed of *H* elements each corresponding to a given hour of the day, and each equal to either 1 (*on*) or 0 (*off*). Inspired by real-world consumption patterns, the initial distribution of *on* elements within the *N* vectors ***θ***_*i*_ is such that their normalized sum has the shape shown in the bottom panel of Fig 4 (light blue bars). The target configuration is now also a vector ***ϵ*** of length *H*, whose shape (lilac bars in Fig 4) is inspired by real-world microgrid production patterns based mostly on solar energy, and we set

$$\begin{array}{c} \sum_{h=1}^{H}\epsilon_{h}=\sum_{i=1}^{N}\sum_{h=1}^{H}\theta_{i,h}\;\text{.} \end{array}$$ (12)

As in the previous model, at each time step an user *i* is randomly chosen and evaluates the local configuration, given by its own and its neighbors’ state vectors:

$$\begin{array}{c} \tau \left(i\right)=\frac{N}{k_{i}+1}\left(\theta_{i,1}+\sum_{n\in N(i)}\theta_{n,1},\theta_{i,2}+\sum_{n\in N(i)}\theta_{n,2},\ldots ,\theta_{i,H}+\sum_{n\in N(i)}\theta_{n,H}\right)\;\text{.} \end{array}$$ (13)

The user then “moves” one of its loads that is in a hour slot *h*′ (i.e. *θ*_*i*,*h*′_ = 1) where the local configuration exceeds the available energy (i.e. $|\epsilon_{h^{\prime }}-\frac{N}{k_{i}+1}\left(1+\sum_{n\in N(i)}\theta_{n,h^{\prime }}\right)\left|>\right|\epsilon_{h^{\prime }}-\frac{N}{k_{i}+1}\sum_{n\in N(i)}\theta_{n,h^{\prime }}|$), to a hour slot *h*″ where currently the user has no load (i.e. *θ*_*i*,*h*″_ = 0), and adding a load would improve the matching between the local and the target configuration (i.e. $|\epsilon_{h^{\prime \prime }}-\frac{N}{k_{i}+1}\sum_{n\in N(i)}\theta_{n,h^{\prime \prime }}\left|<\right|\epsilon_{h^{\prime }}-\frac{N}{k_{i}+1}\left(1+\sum_{n\in N(i)}\theta_{n,h^{\prime \prime }}\right)|$). If this is the case for more than one pair of hour slots (*h*′, *h*″), then the slots are chosen with probability proportional to the improvement that the move would provide to the matching between the target and the local configuration. At the end of this evaluation process, *θ*_*i*,*h*″_ = 1 and *θ*_*i*,*h*′_ = 0, and then a new user is randomly selected, and so on, until the system reaches a stationary state. The two bottom panels of Fig 4 show the results of numerical simulations of the model on ER graphs with different edge probabilities *p*. The left panel shows, for each *p*, the system *coordination level* at the stationary state, now defined as

$$\begin{array}{c} 1-\frac{1}{2\sum_{h=1}^{H}\epsilon_{h}}\sum_{h=1}^{H}\left|\epsilon_{h}-\left(\sum_{i=1}^{N}\theta_{i,h}\right)\right|\;\text{.} \end{array}$$ (14)

The right panel shows the system *alteration level*, defined as the average difference between the users’ state vector at the beginning of the dynamics and at the stationary state, that is:

$$\begin{array}{c} \frac{1}{2N}\sum_{i=1}^{N}\sum_{h=1}^{H}\left|\theta_{i,h}\left(t=\infty \right)-\theta_{i,h}\left(t=0\right)\right|\;\text{.} \end{array}$$ (15)

Again, full coordination and minimal alteration are obviously reached at *p* = 1. The coordination level then decreases as *p* decreases, until a critical point below which coordination starts increasing again, until it drops again for *p* = 0, as found in the previous model too. Interestingly, in ER networks of 1000 nodes, there is a range of *p* values, from *p* = 0.003 to *p* = 0.001, for which coordination increases while alteration decreases. This means that even in this extended model we find that a reduced set of coordinating users can actually be beneficial to approach the target configuration. Therefore, in the microgrid scenario with no complete information on the consumption patterns of all households, having information about, on average, only one other household can lead to a good matching between the grid available energy and the households’ total load, with relatively little alteration in users schedules.

The proposed model clearly presents a series of limitations. First, energy loads are modeled as binary (either *on* or *off*), instead of using numerical values representing consumed kilowatt-hours. Second, the microgrid is assumed to produce sufficient energy to supply the total daily consumption, which is often not the case in real microgrid settings. Third, the role of prosumers is not explicitly taken into account, the energy they produce being implicitly included in the total available energy *E*. These assumptions are strong and need to be overcome in future work. Moreover, the modeled social coordination takes place on top of a physical network—the distributed grid—requiring additional synchronization. Future work should explicitly take into account both layers to attempt at proposing a more comprehensive model of physical and social synchronization in distributed energy systems.
